# Biological Activity of Autochthonic Bacterial Community in Oil-Contaminated Soil

**DOI:** 10.1007/s11270-016-2825-z

**Published:** 2016-04-04

**Authors:** Agnieszka Wolińska, Agnieszka Kuźniar, Anna Szafranek-Nakonieczna, Natalia Jastrzębska, Eliza Roguska, Zofia Stępniewska

**Affiliations:** Institute of Biotechnology, Department of Biochemistry and Environmental Chemistry, The John Paul II Catholic University of Lublin, Konstantynów 1 I, 20-708 Lublin, Poland

**Keywords:** Enzyme activity, Microbial community, Soil contamination, Petroleum derivates substances

## Abstract

Soil microbial communities play an important role in the biodegradation of different petroleum derivates, including hydrocarbons. Also other biological factors such as enzyme and respiration activities and microbial abundance are sensitive to contamination with petroleum derivates. The aim of this study was to evaluate the response of autochthonic microbial community and biological parameters (respiration, dehydrogenase and catalase activities, total microorganisms count) on contamination with car fuels and engine oils. The surface layer (0–20 cm) of *Mollic Gleysol* was used for the experiment. In laboratory conditions, soil was contaminated with the following petroleum substances: car fuels (petrol, diesel) and car engine oils (new and waste—after 10,000 km). The results demonstrated that, among the investigated hydrocarbon substances, petrol addition seemed to be the most toxic for the microbial activity of the investigated soil. The toxicity of the used hydrocarbon substances to microorganisms might be summarized as follows: diesel > new oil > waste oil > petrol. Species belonging to the genera *Micrococcus* and *Rhodococcus* were noted as the major autochthonic bacteria being present in soil contaminated with new automobile oil, whereas species of the genera *Bacillus* sp. and *Paenibacillus* sp. were identified in the combination treated with waste oil.

## Introduction

The intensive development of urbanization and mechanization is associated with increases in the environment contamination by petroleum and petroleum derivate products (Rusin et al. 2015). Oil derivates and car fuels can be regarded as composed of four major constituents: saturated hydrocarbons, aromatic hydrocarbons, asphaltenes, and resins (Moubasher et al. 2015; Ramadass et al. 2015). Many polycyclic aromatic hydrocarbons (PAHs) and their epoxides are highly toxic, mutagenic, or carcinogenic to microorganisms and higher organisms, including humans (Moubasher et al. 2015). Generally, car oil consists of 90 % petroleum fractions and 10 % of other additives, i.e., antioxidants and detergents (Ramadass et al. 2015); however, a particular danger for the soil environment is posed by used oil. The Environmental Protection Agency (2001) defined used motor oil as “any petroleum-based or synthetic oil that has been used for vehicle lubrication and as a result of normal use, motor oil becomes contaminated with various impurities such as dirt, water, chemicals, or metals from vehicle engine”. It was reported that 1 L of used motor oil can pollute up to 3,784 m^2^ of soil, making it non-productive for farming or plant growth for up to 100 years (Chin et al. 2012). In that context, soil contamination with oil derivates is a serious problem needing recognition particularly in the aspect of autochthonic soil microorganisms that are able to survive in such extremely difficult conditions. Soil microorganisms in intimate contact with soil particles are very sensitive to any ecosystem perturbation and are therefore considered to be the best indicators of soil pollution (Andreoni et al. 2004; Guo et al. 2012). Suja et al. (2014) reported that the use of indigenous microorganisms in the bioremediation process can reduce the risk associated with hydrocarbon contamination of soils. Earlier studies indicated that the following soil bacterial genera have a capability of bioremediation of petroleum substances: *Pseudomonas*, *Arthrobacter*, *Acinetobacter*, *Nocardia*, *Corynebacterium*, *Geobacillus*, *Klebsiella*, *Bacillus*, *Mycobacterium, Brachybacterium, Microbacterium, Sphingobium*, and *Serratia* (Al-Mailem et al. 2014; Chen et al. 2015; Rusin et al. 2015). Also, such genera as *Micrococcus* (Khan and Singh 2011), *Pseudomonas* sp. (Nikhil et al. 2013), and *Rhodococcus* (Leilei et al. 2012) are indicated to have an ability to degrade hydrocarbon in the soil environment.

The biological balance in soil affected by the toxicity of petroleum compounds could be assessed by ecotoxicological assays using bacteria (Hentati et al. 2013; Moubasher et al. 2015) or by measuring the activities of soil enzymes, e.g., dehydrogenases and catalase (Wyszkowska and Wyszkowski 2010; Hentati et al. 2013; Alrumman et al. 2015). Soil enzymatic activity assays act as potential indicators of ecosystem quality, as they are operationally practical, sensitive, integrative, and often described as “soil biological fingerprints”. Dehydrogenases (EC 1.1.1.) are particularly important soil enzymes, since they exist only inside viable microbial cells and thus provide reliable information about soil biology, fertility, and productivity (Frąc and Jezierska-Tys 2011; Wolinska et al. 2015). Thus, measurement of dehydrogenase activity (DHA) in soil provides a large amount of information about its biological characteristics. Catalase (EC 1.11.1.6) is another intracellular enzyme found in all aerobic bacteria and most facultative anaerobes, but absent in obligate anaerobes (Shiyin et al. 2004). The products of oxygen reduction, such as hydrogen peroxide, superoxide radical, and hydroxyl radical, can be highly toxic to cells and might damage cellular macromolecules (Stępniewska et al. 2009). Thus, the main catalase function is to split hydrogen peroxide into molecular oxygen and water and thus prevent cells from damage by reactive oxygen species (Shiyin et al. 2004; Yao et al. 2006). It is assumed that catalase activity (CAT) may be related to the metabolic activity of aerobic organisms and has been used as an indicator of soil fertility (Shiyin et al. 2004; Stępniewska et al. 2009). Also, respiration activity (RA), defined as the process of carbon dioxide release by microorganisms and plant roots, is a factor that provides one of the most important information about the soil biological activity (Cerhanova et. al. 2006; Baronti et al. 2008). However, little investigation has been carried out to examine the effect of contamination with petroleum derivates on RA.

It should be emphasized that soil quality can be determined according to the presence and activity of soil microbial populations, whereas enzyme activities have been considered as parameters for evaluating and monitoring remediation of hydrocarbon-contaminated soil (Alrumman et al. 2015). Thus, in the current study, soil dehydrogenase, soil catalase, soil respiration, and the number of cultivable microorganisms were used to monitor the effects of oil derivates on soil quality. Additionally, identification of the microbial community was performed with the use of molecular methods. The purpose of this work was to determine the effect of petroleum-derived substances such as petrol, diesel, and new and used engine oils on selected biological factors and to carry out molecular identification of microorganisms that are able to perform the bioremediation process in contaminated *Mollic Gleysol*.

## Material and Methods

### Soil Characteristic

**Fig. 1:**
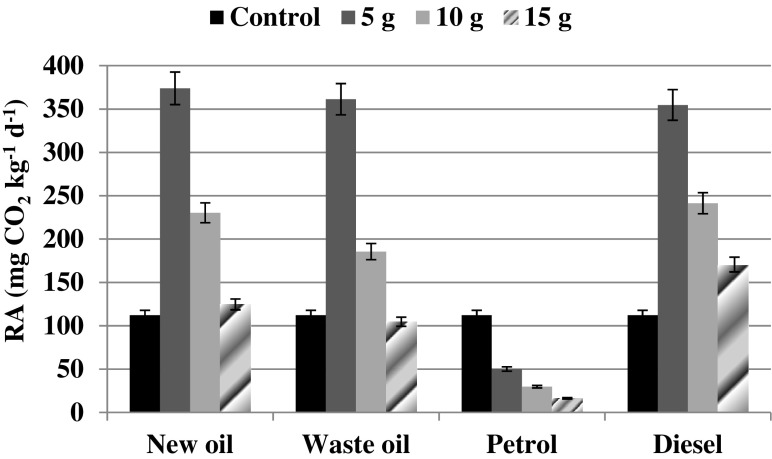
Soil respiration activity at different level of petrol, diesel, and oil derivates contamination. *Error bars* represents standard deviation (*n* = 3)

The soil used for the experiment was *Mollic Gleysol* taken in October 2014 from Kosiorów village (51°13′N; 21°51′E; Fig. 1), located close to the Chodelka River, a tributary of the Vistula River in the south-east part of Poland. Soil was collected from the surface layer (0–20 cm) of an agricultural meadow used for hay-making.

The soil was chemically characterized by analyses of pH, redox potential (Eh), electric conductivity (EC) by Hach Lange and Radiometer potentiometric equipment and total carbon (TC) content by an automatic analyzer Shimadzu TOC-V CSH. The soil investigated exhibited a peaty soil type (upper 20 cm: pH 6.19; Eh 480.73 mV; EC 197.8 μS/cm^3^).

In laboratory conditions, the soil was contaminated with the following petroleum substances: car fuels (unleaded 95-octane petrol and diesel) and car engine oils (synthetic Total 5W/30, referred to as New oil and Mobil 1™ 5W/50 after driving 10,000 km, referred to as Waste oil). Synthetic oil 5W/30 is particularly suited for turbocharged, multi-valve, and direct injection engines. It can be used in the most severe operating conditions in any type of climate. It is perfectly adapted to all vehicles equipped with catalysts that use unleaded fuel or LPG. Mobil 1™ is the world’s leading synthetic motor oil brand delivering ultimate car engine performance and protection. The balanced technology of Mobil 1 5W/50 helps to make it suitable for many types of vehicles and operating conditions, from mild to severe. Car fuels (petrol, diesel) and engine oils (new and waste) were added to the soil at the following doses: 5.0, 10.0, and 15.0 g per 10 g of soil. Soil samples without any petroleum substances addition were prepared as a control. Prior to laboratory assays, the contaminated and control soils were incubated for 1 week at room temperature (20 °C).

### Soil Respiration Activity

Soil respiration activity (RA) was determined with the use of a gas chromatograph (GC Varian CP-3800, USA) equipped with a TCD detector and two types of columns: Poraplot Q (25 m) and a molecular sieve 5A (30 m) connected together (Szafranek-Nakonieczna and Stępniewska 2014). Soil subsamples (10 g, three replicates, both control and contaminated treatments) were placed in dark, sterile bottles (60 ml) tightly closed and 1-week incubated (20 °C). At the beginning of the experiment and after 7 days, the level of accumulated CO_2_ was analyzed in the headspace of the soil samples. Based on the differences between the concentration of CO_2_ at the start and at the end of the experiment, RA was calculated and expressed as a mass of produced carbon dioxide per mass of dry soil and per unit of time—day (milligram of CO_2_ per kilogram of d.m. per day).

### Assay for Determination of Soil Enzymatic Activities

Soil dehydrogenase activity (DHA) was estimated by reducing 2.3.5–triphenyltetrazolium chloride (TTC), according to the procedure of Casida et al. (1964). Soils samples (6 g, three replicates, both control and contaminated treatments) were mixed with 120 mg CaCO_3_, 1 ml 3 % (*w*/*v*) TTC, and 4 ml of distilled water, and incubated for 20 h at 30 °C (Heraens Instruments). Then, extraction with ethanol (25 ml) was performed. After 1-h incubation in the dark, the extracts were filtered and absorption was measured at 485 nm (UV-1800, Shimadzu).

Catalase activity (CAT) was determined by back-titrating residual H_2_O_2_ with KMnO_4_ according to the Johnson and Temple (1964) method. Soil samples (2 g, three replicates, both control and contaminated treatments) were added to 40 ml distilled water with 5 ml of a 0.3 % hydrogen peroxide solution. The mixture was shaken for 20 min and then 5 ml of 1.5 M H_2_SO_4_ were added. Afterwards, the solution was filtered and titrated using 0.02 M KMnO_4_ (Stępniewska et al. 2009). The reacting amount of 0.02 M KMnO_4_ calculated per gram of dry soil was used to express the activity of catalase.

### Number of Soil Microorganisms

The total microbial counts (TMC) were estimated by the viable count on serial spread plates (Guo et al. 2012). The soil samples (5 g, three replicates, both control and contaminated treatments) were suspended separately in 50 ml 0.9 % NaCl. The series of dilutions were repeated to produce six continuous dilutions. The c.f.u. (colony-forming unit) of the total heterotrophic bacteria was counted after 14 days of growth on an agar medium at 25 °C (Wolińska et al. 2013).

### Isolation of Petroleum-Degrading Bacterial Strains

Petroleum-contaminated and control soil samples (5 g) were added to a flask containing 50 ml of 0.9 % NaCl as 10^−4^ diluents, which were then shaken for 30 min at 150 rpm at 30 °C. Then, 0.1 ml was loaded onto LB solid medium plates and incubated for 7 days at 30 °C. Microbial colonies with different color and form were transferred with an inoculation loop onto solid LB medium again. This step was repeated twice for separation and purification. The purified strains were precultivated two times on enrichment liquid medium and were used as petroleum-degrading bacteria for further study. Then, the morphological characteristics of the colonies were observed, e.g., colony color, form, and Gram staining.

### Molecular Identification

The total bacterial DNA was extracted with the Sambrook and Russell method (2001, with own modification). The universal 16S rDNA primers 27F (5′-AGAGTTTGATCATGGCTCAG-3 ′) and 1492 R (5′-TACGGTTACCTTGTTACGACTT-3′) were synthesized to amplify the 16S rRNA gene of the isolates obtained, using its genomic DNA as a template. For the PCR reaction (50 μl), 1X Phusion Flash High-Fidelity PCR Master Mix (Thermo Scientific) was used. The reaction conditions consisted in initial denaturation at 98 °C for 10 s, 30 cycles of 98 °C for 5 s, primer annealing at 53 °C for 5 s, and elongation at 72 °C for 40 s. Afterwards, the PCR amplification products were separated by electrophoresis in a 1 % agarose gel. Then, all products were gel purified and sequenced. The 16S rDNA fragments of all isolates obtained were sent to the Genomed S.A. (Warsaw) for sequencing. The results obtained were analyzed by BLAST online comparison (http:// www.ncbi.nlm.nih.gov) for identification of the isolates.

## Results

### Effect of Car Fuels and Engine Oils on Soil Respiration Activity

The response of RA on soil contamination with petroleum substances after 1-week incubation is shown at Fig. 1. RA registered in the control sample was 112.25 mg CO_2_ kg^−1^ day^−1^. The addition of oil derivates and diesel at every dose applied resulted in RA stimulation in comparison to respiration from the control soil. Three-fold higher values of RA ranging between 355 and 378 mg CO_2_ kg^−1^ day^−1^were noted after 5.0 g of contaminant addition in regard to diesel as well as new and waste oil. The dose of 10.0 g caused a double increase in RA, which persisted at 241, 230, and 185 mg CO_2_ kg^−1^ day^−1^ for diesel, new oil, and waste oil, respectively. Also, the highest contaminant dose caused c.a. 30 % RA stimulation in relation to the control value. However, the strongest effect was observed in the case of 15.0 g diesel application when RA amounted to 170 mg CO_2_ kg^−1^ day^−1^.

Among the investigated hydrocarbon substances, petrol addition seemed to be the most toxic to the microbial activity expressed as RA, as a significant (*p* < 0.001) decrease in respiration, irrespective of the petrol dose, was observed. RA decreased by 55, 73, and 85 % in comparison to the control value as an effect of the following petrol doses: 5.0, 10.0, and 15.0 g.

### Effect of Car Fuels and Engine Oils on Soil Enzymatic Activity

**Fig. 2:**
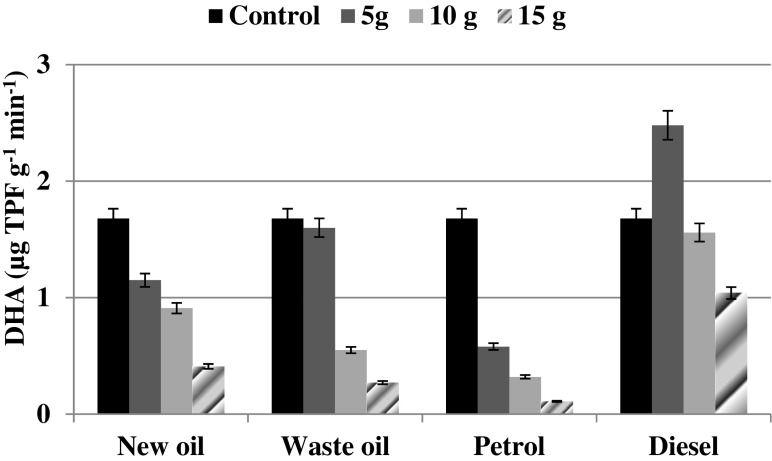
Soil dehydrogenase activity at different level of petrol, diesel, and oil derivates contamination. *Error bars* represents standard deviation (*n* = 3)

The response of DHA on soil contamination with petroleum substances is presented at Fig. 2. DHA detected in the non-contaminated soil samples had a value of 1.68 μg TPF g^−1^ min^−1^. Stimulation of DHA was observed only in one case as a result of diesel (5.0 g) addition when DHA equaled 2.48 μg TPF g^−1^ min^−1^. In the other cases, the addition of 5.0 g of petroleum substances led to a decrease in DHA in comparison to the control value by 4.8, 31.5, and 65.5 % for waste oil, new oil, and petrol, respectively. The higher oil and petrol doses (10.0 and 15.0 g) resulted in a further DHA decline, regardless of the type of oil products. The lowest level of DHA of 0.11–0.41 μg TPF g^−1^ min^−1^ was detected in the soil samples contaminated with 15.0 g of oil products. However, DHA has proved to be the most resistant to diesel pollution, as even the highest dose (15.0 g) resulted in 38 % reduction of enzymatic activity in comparison to the control value.

**Fig. 3:**
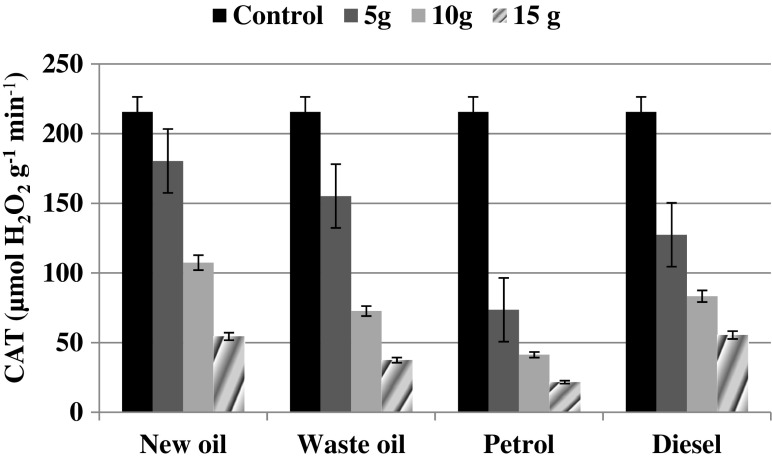
Soil catalase activity at different level of petrol, diesel, and oil derivates contamination. *Error bars* represents standard deviation (*n* = 3)

Also, CAT turned out to be sensitive to contamination with petrol, diesel, and oil derivates as each of the contaminant doses applied induced a decrease in CAT (Fig. 3). CAT recorded in the control samples amounted to 216.61 μmol H_2_O_2_ g^−1^ min^−1^. The lowest contaminant dose (5.0 g) led to CAT reduction by c.a. 16–66 % in comparison to the non-contaminated soil. The most toxic effect in this case was posed by petrol, whereas new oil and diesel seemed to be the least harmful to CAT.

### Effect of Car Fuels and Engine Oils on the Number of Soil Microorganisms

**Fig. 4:**
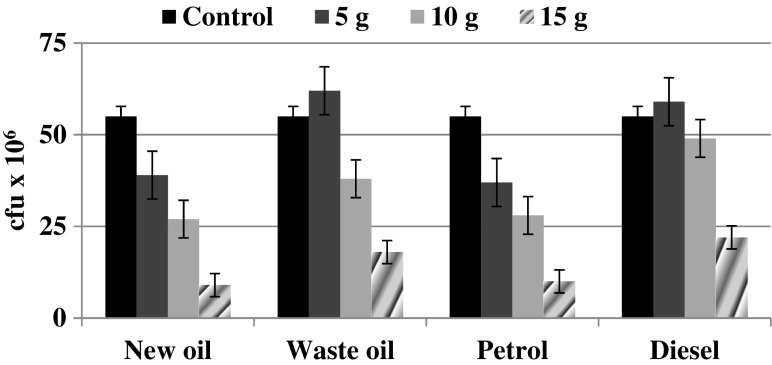
Soil microorganisms number at different level of petrol, diesel, and oil derivates contamination. *Error bars* represents standard deviation (*n* = 3)

The changes noted in the microorganisms abundance caused by contamination with petrol, diesel and oil derivates and expressed as colony forming units are demonstrated at Fig. 4.

Cultivable microorganisms number (CMN) noted in the control samples reached the level of 55 × 10^6^ c.f.u. In two cases, addition of diesel and waste oil (5.0 g) resulted in an increase in the bacterial abundance by 7 and 13 %, respectively. However, the differences mentioned were not significant (*p* > 0.05). Each subsequent dose of the contaminants caused a decline in CMN by 35 and 73 % in respect to the contaminant dose: 10.0 and 15.0 g. However, it should be emphasized that despite the use of the highest doses of the petroleum substances (15.0 g), CMN was relatively high and ranged between 9 and 22 × 10^6^ c.f.u., which suggests therefore that the bacterial microflora inhabiting the investigated *Mollic Gleysol* is capable of growth in the presence of contamination with petroleum substances. Consequently, the next step in the study was molecular identification of the autochthonic microbial community.

### Effect of Car Fuels and Engine Oils on Soil Bacterial Community

**Fig. 5:**
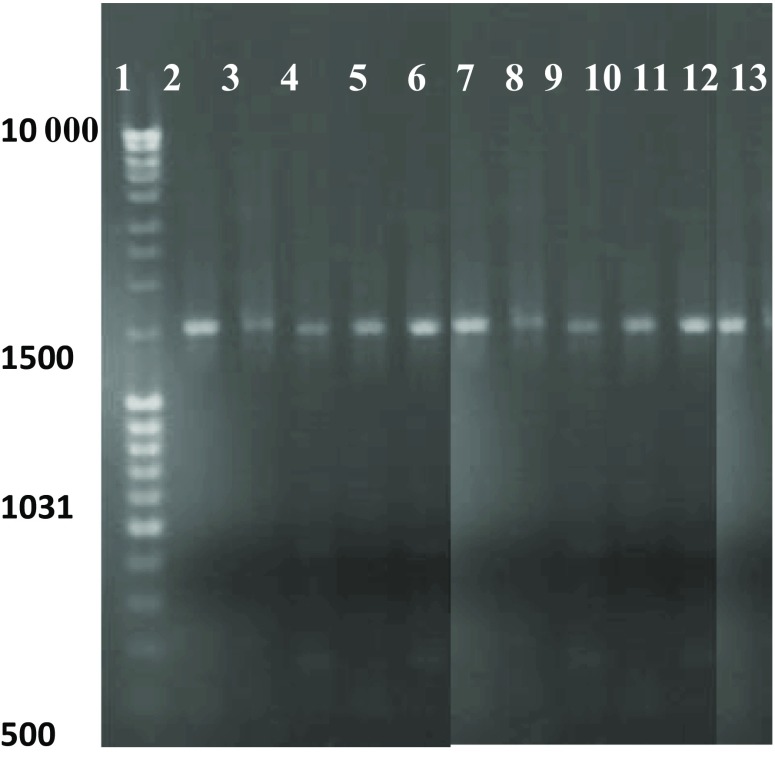
Agarose gel electrophoresis of PCR products (gene fragment 16S rRNA, 1,500 kb): *Line 1*—molecular mass marker DNA, *2*—C1, *3*—C2, *4*—WO1, *5*—WO2, *6*—NO1, *7*—NO2, *8*—P1, *9*—P2, *10*—D1, *11*—D2, *12*—positive control, *13*—negative control

We isolated 10 strains from the top soil layer (both control and contaminated soil treatments). The DNA from the bacterial isolates obtained was the template for PCR reaction with universal 16S rDNA primers 27F/1492R. We obtained specific PCR amplification products ca. 1.5 Kb in size (Fig. 5). All the PCR fragments were highly similar to known bacterial strains according to the 16S rDNA sequence comparison.

The major autochthonic bacteria present in the soil contaminated with petrol oil and diesel as well as the control soil included species belonging to the genera *Rhodococcus* (*R. erythropolis*, *R. qingshengi*, and *R. globerulus*)—isolates C2, P1, P2, D1, and D2, whereas isolates C1, WO1, WO2, NO1, and NO2 representing the genera *Micrococcus*, *Bacillus*, *Peanibacillus*, *Mesorhizobium* (Table 1) were identified in the combination treated with waste and new engine oil.

**Table 1:** Identification of isolates based on 16S rDNA sequencing data, abbreviations of strains isolated from control soil—C1, C2, strains isolated from soil amended, respectively WO1, WO2—waste engine oil; NO1, NO2—new engine oil; B1, B2—petroleum; D1, D2—diesel

Isolate code	Identification result		Similarity (%)	No. of GenBank	Source	Reference
C1	*Micrococcus* sp.	*Micrococcus* sp. CNJ719 PL04	95	DQ448712	Marine sediments	Gontang et al. 2007
		*Micrococcus* sp. 76A3b	95	KJ744024	Soil	Gontang et al. 2007
C2	*Rhodococcus* sp.	*Rhodococcus* sp. A10	98	JQ976987	Bulk soil	Song and Sheng 2013
		*Rhodococcus qingshengii* strain B2	98	KJ028076	Sludge	Kim 2014
WO1	*Bacillus* sp.	*Bacillus* sp. WYT001	95	JQ807851	Soil	Deng and Wen 2012
		*Bacillus anthracis* strain IHB B 7021	96	KJ721202	Rhizosphere	Gulati et al. 2015
WO2	*Paenibacillus* sp.	*Paenibacillus* sp. HD1PAH	92	KM587692	Oil-contaminated soil	Deka and Lahakar 2014
		*Paenibacillus glucanolyticus* strain BGR23	92	KC789782	Soil	Zhang 2013
		*Paenibacillus vortex strain* KSI 323	92	KC113142	Soil	Hashemi et al. 2012
NO1	*Micrococcus* sp.	*Micrococcus* sp.	95	KJ843153	Onion waste	Rinland and Gomez 2014
NO2	*Mesorhizobium* sp.	*Mesorhizobium* sp. E14	95	AB265160	Subsurface soil of the Atacama Desert	Lester et al. 2007
		*Mesorhizobium* sp. SBB-POLY	95	KC521436	Endophytes of *Meganerilla bactericola*	Summers et al. 2013
		*Mesorhizobium* sp. E11	95	AB265157	Subsurface soil of the Atacama Desert	Lester et al. 2007
P1	*Rhodococcus sp.*	*Rhodococcus sp. Poz54*	98	KM670434	Oil-polluted soil	Ortega-Gonzalez et al. 2015
		*Rhodococcus qingshengii strain* PT2-14B	98	KF360060	Oil-polluted hydrocarbons	Tancsics et al. 2014
		*Rhodococcus erythropolis* strain VOL	98	KF499507	Soil	Yolanda et al. 2013
P2	*Rhodococcus sp.*	Uncultured *Rhodococcus* sp. clone SQ8_Pitesti	96	KM282585	Oil-polluted soil	Nohit et al. 2007
		*Rhodococcus* sp. Y2-2-10	96	DQ366088.1	Oil-polluted soil (fuel station)	Van Pham et al. 2014
		*Rhodococcus* sp. BF-5(2014)	96	KM282585	Soil	Wang 2014
D1	*Rhodococcus sp.*	*Rhodococcus erythropolis* strain HS9	96	AY168587	Oil-polluted soil	Seth-Smith et al. 2005
		*Rhodococcus* sp. 3/1(2007)	96	EU041709	Soil	Aislabie et al. 2008
		*Rhodococcus* sp. DSD51Y	96	AB847903	Soil	Ding et al. 2013
D2	*Rhodococcus sp.*	*Rhodococcus qingshengii* strain AF1	97	KM873626	Oil-polluted hydrocarbons	Ferreira et al. 2014
		*Rhodococcus erythropolis* strain NR_48	97	KM113031	Oil-polluted petroleum	Zhao 2014
		*Rhodococcus sp.* Poz54	97	KF747345	Oil-polluted petroleum	Ortega-Gonzalez et al. 2015

Our study reports a preliminary identification. Further identification to the species level is needed. The results of Gram staining and genetic identification showed that nine among the ten isolates are Gram-positive microorganisms. This could be the premise that Gram positive microorganisms are more able to withstand contamination with petroleum (Table 2). The colony shape was established as round or irregular (for most isolated strains, 90 %). The isolated colonies of the strains exhibited mostly a rod shape. As for the colony color or transparency/opacity, it was noticed that most strains formed opaque colonies, weakly pigmented (only one colony of strains was bright yellow), characteristics of microorganisms present in the soil (Table 2).

**Table 2:** Morphological characteristic of obtained isolates (abbreviations explained in Table 1)

Isolates	C1	C2	WO1	WO2	NO1	NO2	P1	P2	D1	D2
Features										
Colony form	Irregular	Irregular	Irregular	Irregular	circular	Irregular	Irregular	Irregular	Irregular	Irregular
Colony color	White	White	White	Bright yellow	White	White	White/cream	White	White/cream	White
Cell shape	Spherical	Rod	Rod	Rod	Rod	Spherical	Rod	Rod	Rod	Rod
Gram staining	+	+	+	+	−	+	+	+	+	+

## Discussion

Enzyme activity could be a good indicator of soil quality because it is sensitive and reflects the biological situation in the soil (Wyszkowska and Wyszkowski 2010; Wolińska et al. 2015). Thus, in the current study, we established soil biological activity by measuring DHA and CAT, and also other contamination-sensitive soil parameters such as RA and CMC. The results obtained confirm that soil enzyme activity is strongly determined by the degree of petrol and oil pollution.

Addition of petroleum substances to the soil was both stimulatory and inhibitory to AR, DHA, CAT, and CMC depending on the nature and concentration of the car fuels and engines oil. We found that addition of car fuels (petrol, diesel) and engine oil (new and waste) up to 5.0 g was usually not toxic to the overall microbial activity since hydrocarbons present in oil could be serving as an additional carbon source for microbial growth (Alrumman et al. 2015; Rusin et al. 2015). Moreover, another cause of the increased soil biological activity might be the ongoing biodegradation process carried out with the participation of autochthonic microorganisms. Achuba and Peretiemo-Clarke (2008) observed progressive enhancement in soil DHA and CAT relative to the control, where concentrations of the spent motor oil increased from 1 to 2 %. Similarly, Ramadass et al. (2015) clearly indicated that even short-term exposure of soils to motor oils leads to changes in soil enzyme activities. In the case of fresh oil at low concentrations, a significant increase in DHA was noted while higher doses resulted in a decrease in enzyme activity. A decreasing trend of DHA at a high concentration of hydrocarbons was also observed by Alurmman et al. (2015).

Among the investigated hydrocarbon substances, petrol addition seemed to be the most toxic for microbial activity of the investigated *Mollic Gleysol*. The toxicity of the used hydrocarbon substances to microorganisms inhabiting the investigated soil type might be summarized as follows: diesel > new oil > waste oil > petrol. Ramadass et al. (2015) suggested that used oils are the most hazardous mainstream categories of environmental pollutants, posing a major threat to the environment and public health because they are responsible for immobilization of nutrients and lowering of soil pH (Shukry et al. 2013). However, this partially remains in contrast to our results, as we observed a less toxic effect posed by waste oil rather than by new oil in relation to DHA and CMC. In that case, even 13 % stimulation of those biological factors resulting from waste oil addition was noted in relation to new oil. On the other hand, the diesel treatment was characterized by higher values of all measured biological factors and this may be due to the fact that diesel is a less toxic source of carbon for microorganisms. A similar finding was reported by Alrumman et al. (2015) in regard to microbial biomass and enzyme activity (dehydrogenases and phosphatases). Measuring soil enzymatic activities can provide information about the function and structure of soil microbial communities in hydrocarbon-contaminated soils. These measures could be used as rapid and cost-effective means for evaluating and monitoring remediation of hydrocarbon-contaminated soil (Alrumman et al. 2015).

For plate counts, it is known that microbial populations proliferate following addition of readily assimilated substrates to the medium (Alrumman et al. 2015). Thereby hydrocarbons in soil provide a source of C for microbial growth and this explains the high c.f.u. for bacteria in the freshly contaminated soils compared to the control soil. This fact explains the 7–13 % increase in the bacterial abundance observed in the current study as an effect of diesel and waste oil addition (5.0 g). Our results are also compatible with those of Guo et al. (2012), who observed that the cultivable microbial count was significantly higher in the low petroleum pollution than in the medium- and high-pollution groups. This may be caused by the use of low toxicity petroleum hydrocarbons as a carbon and energy source by soil microorganisms adapted at a lower dose (Guo et al. 2012).

Other studies have reported that an increase in the number of hydrocarbon utilizers is positively correlated with the hydrocarbon concentration (Margesin et al. 2000; Alrumman et al. 2015). Additionally, the increase in the number of cultivable hydrocarbon-degrading bacteria demonstrates how rapidly indigenous soil microorganisms are able to adapt to new substrates (Margesin et al. 2000).

Based on the morphological (Table 2) and genetical results, it was shown that strains C1 and NO1 belong to the genus *Micrococcus*. Khan and Singh (2011) presented that *Micrococcus varians* can be a potent source for remediation of oil-contaminated sites and can be beneficial for the environment. Earlier studies showed that isolated strain identified tentatively as *M. varians* have the roles of *M. varians* in hydrocarbon bioremediation (Ijah and Antai 2003; Ekpo and Udofia 2008). Modified diesel engine oil medium was used for isolation of oil-degrading strains by Nikhil et al. (2013). The most abundant microorganisms were isolated from garage soil—*Micrococcus* sp. and *Pseudomonas* sp. (Nikhil et al. 2013). The studies reported by Kumar et al. (2013) indicated the application of *Micrococcus* isolated from the contaminated sites to efficiently degrade the crude oil components. The rate of oil degradation by *Micrococcus* sp. has been estimated at ca. 27 % in optimum conditions. Recently, Jesubunmi (2014) has reported that the best bacterial spent oil-degraders were *Micrococcus* species, in comparison to *Aspergillus* and *Penicillium* species of fungi.

Strains C2, P1, P2, D1, and D2 were tentatively identified as *Rhodococcus* species. Strain 3C-9 of *Rhodococcus erythropolis* was identified by Peng et al. (2007) as a candidate for use in oil spill cleanup operations. Leilei et al. (2012) isolated a *Rhodococcus* strain with oil-degrading potential from activated sludge in an oil field. The results of Benedek et al. (2012) indicated that *Rhodococcus qingshengii* BBG1 is suitable for elimination of aliphatic, monoaromatic, and polycyclic aromatic hydrocarbons from soil samples. In studies performed by Van et al. (2014), it was demonstrated that *R. jialingiae* Y1-1 has relatively high oil-degradation efficiencies (>70 %) in land farming, especially during winter.

Strain WO2 was identified as a *Paenibacillus* sp. These bacteria are very interesting, as they are microorganisms with no genomic information. Nowadays, researchers are working on more extensive genome sequencing of *Paenibacillus* sp. which could provide fundamental insights into pathways involved in the complex social behavior of these bacteria and contribute to a discovery of a rich source of genes with biotechnological potential. In literature, there are some reports of the oil-degrading ability of *Peanibacillus* species. Ganesh and Lin (2009) isolated three strains D2, D9, and D10 that were identified as *Paenibacillus* sp. All isolates were capable of degrading 70–80 % of n-paraffin. This study clearly demonstrates that Gram-positive biosurfactant-producing bacteria are effective in diesel degradation (Genesh and Lin 2009).

## Conclusions

In a summary, soil biological analyses such as AR, DHA, CAT, and CMC can shed light on the presence of existing microorganisms in *Mollic Gleysol* contaminated by petroleum substance. In regard to car engine oils, we demonstrated that waste oil (after 10,000 km) was less harmful to soil biology than new oil, which was confirmed by the higher values (by c.a. 44 %) of TMC and CAT. The CAT activity assays showed that the catalase in the studied aerobic autochthonic community could be inductively expressed in the presence of new oil.

Others factors (RA, DHA) remained at a similar level regardless of whether the soil was contaminated by new or waste oil. The lowest dose of the contaminants (5.0 g) resulted even in stimulation of RA, DHA, CAT, and TMC in relation to the control samples.

In regard to car fuels, it was indicated that petrol is more harmful to soil biology than diesel, which was confirmed by the lower values (by c.a. 70 %) of each biological factor (RA, DHA, CAT, MA) treated with petrol in relation to the same diesel contamination doses. RA and DHA seemed to be the most sensitive to petroleum contamination.

Species belonging to the genera *Micrococcus* and *Rhodococcus* were noted as the major autochthonic bacteria being present in soil contaminated with new automobile oil, whereas species of the genera *Bacillus* sp. and *Paenibacillus* sp. were identified in the combination treated with waste oil. Our studies enabled to isolated the strains of bacteria which could be used for oil spills as a microbial component of the biopreparates or the bioaugumentation.

Further studies are very promising because the use of isolated has the following advantages:

- the strains are aerobic microorganisms,
- easy adaptive to new environmental/culture conditions (it is a indigenous flora),
- the use of natural, non-toxic microorganisms,
- avoidance of the use of genetic engineering to increase oil degrading, due to the high flexibility of the metabolism (especially introducing to a genome of a mobile genetic elements)

The impulse to undertake a study on isolation and determination of autochthonous microbiota is the industrial demand for natural, cheap and safe for humans and animals biopreparates.

## References

[CR1] Achuba F, Peretiemo-Clarke B (2008). Effect of spent engine oil on soil catalase and dehydrogenase activities. International Agrophysics.

[CR2] Aislabie J, Ryburn J, Sarmah A (2008). Hexadecane mineralization activity in ornithogenic soil from Seabee Hook, Cape Hallett, Antarctica. Polar Biology.

[CR3] Al-Mailem DM, Kansour MK, Radwan SS (2014). Bioremediation of hydrocarbons contaminating sewage effluent using man-made biofilms: effects of some variables. Applied Biochemistry and Biotechnology.

[CR4] Alrumman SA, Standing DB, Paton GI (2015). Effect of hydrocarbon contamination on soil microbial community and enzyme activity. Journal of King Saud University Science.

[CR5] Andreoni V, Cavalca L, Rao MA, Nocerino G, Bernasconi S, Dell’Amico E, Colombo M, Gianfreda L (2004). Bacterial communities and enzyme activities of PAHs polluted soils. Chemosphere.

[CR6] Baronti S, Tognetti R, Lanini GM, Tonon G, Raschi A (2008). Soil respiration and microbial activity in a Mediterranean grassland expose to free air CO_2_ enrichment (FACE). Community Ecology.

[CR7] Benedek T, Máthé I, Salamon R, Rákos S, Pásztohy Z, Márialigeti K, Lányi S (2012). Potential bacterial soil inoculant made up by *Rhodococcus* sp. and *Pseudomonas* sp. for remediation in situ of hydrocarbon and heavy metal polluted soils. Studia Universitatis Babes-Bolyai Seria Chemia.

[CR8] Casida L, Johnson J, Klein D (1964). Soil dehydrogenase activity. Soil Science.

[CR9] Cerhanova D, Kubat J, Novakova J (2006). Respiration activity of the soil samples from the long-term field experiments in Prague. Plant, Soil and Environment.

[CR10] Chen M, Xu P, Zeng G, Yang C, Huang D, Zhang J (2015). Bioremediation of soil contaminated with polycyclic aromatic hydrocarbons, petroleum, pesticides, chlorophenols and heavy metals by composting: applications, microbes and future research needs. Biotechnology Advances.

[CR11] Chin S, Shafiq N, Nuruddin M (2012). Effects of used engine oil in reinforced concrete beams: the structural behaviour. World Academy of Science, Engineering and Technology.

[CR12] Deka, H., & Lahakar, J. (2014). *Biodegradation of anthracene by Paenibacillus sp. HD1PAH supplemented with biosurfactant of Pseudomonas aeruginosa H7h*. Abstract book of the Third International Symposium on Bioremediation and Sustainable Remediation Technologies. Miami, Florida, p. 210.

[CR13] Deng, A., & Wen, T. (2012). Department of Industrial Microbiology and Biotechnology, Institute of Microbiology. Unpublished.

[CR14] Ding, L.X., Jin, Y., Zhai, J.Y., & Ma, Y.C. (2013). The VBNC bacterium isolated from soil and wastewater by using Rpf. Unpublished.

[CR15] Ekpo MA, Udofia VS (2008). Rate of biodegradation of crude oil by microorganism isolated from oily sludge environment. African Journal of Biotechnology.

[CR16] Ferreira, A.M., Queiros, D., Gagliano, M.C., Serafim, L.S., & Rossetti, S. (2014). Isolation and characterization of PHAs-accumulating bacteria from HSSL. Unpublished.

[CR17] Frąc M, Jezierska-Tys S (2011). Agricultural utilisation of dairy sewage sludge: its effect on enzymatic activity and microorganisms of the soil environment. African Journal of Microbiology Research.

[CR18] Ganesh A, Lin J (2009). Diesel degradation and biosurfactant production by Gram-positive isolates. African Journal of Biotechnology.

[CR19] Gontang EA, Fenical W, Jensen PR (2007). Phylogenetic diversity of gram-positive bacteria cultured from marine sediments. Applied and Environmental Microbiology.

[CR20] Gulati, A., Sood, S., & Thakur, R. (2015). Plant pathology and microbiology lab, HATS Division, CSIR-Institute of Himalayan Bioresource Technology. Unpublished.

[CR21] Guo H, Yao J, Cai M, Qian Y, Guo Y, Richnow HH, Blake RE, Doni S, Ceccanti B (2012). Effects of petroleum contamination on soil microbial numbers, metabolic activity and urease activity. Chemosphere.

[CR22] Hashemi, S.A., Goodarzi, G., & Khodarahmi, F. (2012). Infectious and Tropical Diseases Research Center, Ahvaz Jundishapur University of Medical Sciences, Golestan, Ahvaz 6135715794, Iran. Unpublished.

[CR23] Hentati O, Lachhab R, Ayadi M, Ksibi M (2013). Toxicity assessment for petroleum-contaminated soil using terrestrial invertebrates and plant bioassays. Environmental Monitoring and Assessment.

[CR24] Ijah UJJ, Antai SP (2003). Removal of Nigerian light crude oil in soil over 12-month period. International Biodeterioration and Biodegradation.

[CR25] Jesubunmi CO (2014). Isolation of oil-degrading microorganisms in spent engine oil-contaminated soil. Journal of Biology, Agriculture and Healthcare.

[CR26] Johnson JI, Temple KL (1964). Some variables affecting the measurement of catalase activity in soil. Soil Science Society of America Proceedings.

[CR27] Khan JA, Singh S (2011). Evaluation of oil degradation potential of *Micrococcus varians*. International Journal of Applied Biology and Pharmaceutical Technology.

[CR28] Kim, A.L. (2014). Department of Life Science and Genetic Engineering, Pai Chai University. Unpublished.

[CR29] Kumar KS, Dhanarani TS, Thamaraiselvi K (2013). Utilization of petroleum hydrocarbons by *Micrococcus* and *Streptococcus* spp. isolated from contaminated site. Journal of Microbiology and Biotechnology Research.

[CR30] Leilei Z, Mingxin H, Suiyi Z (2012). Enzymatic remediation of the polluted crude oil by *Rhodococcus*. African Journal of Microbiology Research.

[CR31] Lester ED, Satomi M, Ponce A (2007). Microflora of extreme arid Atacama Desert soils. Soil Biology and Biochemistry.

[CR32] Margesin R, Zimmerbauer A, Schinner F (2000). Monitoring of bioremediation by soil biological activities. Chemosphere.

[CR33] Moubasher HA, Hegazy AK, Mohamed NH, Moustafa YM, Kabiel HF, Hamad AA (2015). Phytoremediation of soils polluted with crude petroleum oil using *Bassia scoparia* and its associated rhizosphere microorganisms. International Biodeterioration and Biodegradation.

[CR34] Nikhil T, Deepa V, Rohan G, Satish B (2013). Isolation characterization and identification of diesel engine oil degrading bacteria from garage soil and comparison of their bioremediation potential. International Research Journal of Environmental Sciences.

[CR35] Nohit, A.M., Vassu, T., Smarandache, D., Csutak, O., Ionescu, R., Soare, S., Ghindea, R., & Stoica, I. (2007). Genomic Analysis on an Oil Degrading Bacterial Consortium. Unpublished.

[CR36] Ortega-Gonzalez DK, Martinez-Gonzalez G, Flores CM, Zaragoza D, Cancino-Diaz JC, Cruz-Maya JA, Jan-Roblero J (2015). *Amycolatopsis* sp. Poz14 isolated from oil-contaminated soil degrades polycyclic aromatic hydrocarbons. International Biodeterioration and Biodegradation.

[CR37] Peng, F., Liu, Z., Wang, L., & Shao, Z. (2007). An oil-degrading bacterium: Rhodococcus erythropolis strain 3C-9 and its biosurfactants. *Journal of Applied Microbiology, 102*(6), 1603–1611. 10.1111/j.1365-2672.2006.03267.x 17578426

[CR38] Ramadass K, Megharaj M, Venkateswarlu K (2015). Ecological implication of motor oil pollution: earthworm survival and soil health. Soil Biology and Biochemistry.

[CR39] Rinland, M.E., & Gomez, M. (2014). Isolation and characterization of onion degrading bacteria from onion waste produced in South Buenos Aires province, Argentina. Unpublished. 10.1007/s11274-015-1803-8 25586510

[CR40] Rusin M, Gospodarek J, Nadgórska-Socha A (2015). The effect of petroleum-derived substances on the growth and chemical composition of *Vicia faba* L. Polish Journal of Environmental Studies.

[CR41] Sambrook J, Russell DW (2001). Molecular cloning: a laboratory manual, 3.

[CR42] Seth-Smith, H.M.B., & Bruce, N.C. (2005). Identification and characterization of RDX degrading bacteria. Unpublished.

[CR43] Shiyin L, Lixiao N, Panying P, Cheng S, Liansheng W (2004). Effects of pesticides and their hydrolysates on catalase activity in soil. Bulletin of Environmental Contamination and Toxicology.

[CR44] Shukry W, Al-Hawas G, Al-Moaikal R, El-Bendary M (2013). Effect of petroleum crude oil on mineral nutrient elements, soil properties and bacterial biomass of the rhizosphere of jojoba. British Journal of Environment and Climate Change.

[CR45] Song, C., & Sheng, H. (2013). Diversity of plant-associated bacteria within subnival plants. Unpublished.

[CR46] Stępniewska Z, Wolińska A, Klin R (2009). Response of soil catalase activity to chromium contamination. Journal of Environmental Science.

[CR47] Suja F, Rahim F, Taha MT, Hambali N, Razali R, Khalid A, Hamzah A (2014). Effects of local microbial bioaugumentation and biostimulation on the bioremediation of total petroleum hydrocarbons (TPH) in crude oil contaminated soil based on laboratory and field observations. International Biodeterioration and Biodegradation.

[CR48] Summers MM, Katz S, Allen EE, Rouse GW (2013). Association of rhizobia with a marine polychaete. Environmental Microbiology Reports.

[CR49] Szafranek-Nakonieczna A, Stępniewska Z (2014). Aerobic and anaerobic respiration in profiles of Polesie Lubelskie peatlands. International Agrophysics.

[CR50] Tancsics A, Benedek T, Farkas M, Mathe I, Marialigeti K, Szoboszlay S, Kukolya J, Kriszt B (2014). Sequence analysis of 16S rRNA, gyrB and catA genes and DNA-DNA hybridization reveal that *Rhodococcus jialingiae* is a later synonym of *Rhodococcus qingshengii*. International Journal of Systematic and Evolutionary Microbiology.

[CR51] US EPA (2001). RCRA Superfund & EPCRA Call Centre Training Module on Introduction to Used Oil.

[CR52] Van Pham HT, Kim J, Jeong SW (2014). Enhanced isolation and culture of highly efficient psychrophilic oil-degrading bacteria from oil-contaminated soils in South Korea. Journal of Environmental Biology.

[CR53] Wang, G. (2014). Aerobic degradation of buprofezin via novel degradation intermediates by a novel isolated *Rhodococcus* sp. BF-5 isolated from buprofezin-contaminated soil. Unpublished.

[CR54] Wolińska A, Stępniewska Z, Kuźniar A (2013). Characterization of microbial community in the selected Polish mineral soils after long term storage. African Journal of Microbiology Research.

[CR55] Wolińska A, Stępniewska Z, Pytlak A (2015). The effect of environmental factors on total soil DNA content and dehydrogenase activity. Archives of Biological Science.

[CR56] Wyszkowska J, Wyszkowski M (2010). Activity of soil dehydrogenases, urease, and acid and alkaline phosphatases in soil polluted with petroleum. Journal of Toxicology and Environmental Health.

[CR57] Yao, X., Min, H., & Yuan, H. (2006). Influence of acetampirid on soil enzymatic activity and respiration. *European Journal of Soil Biology, 42*(2), 120–126

[CR58] Yolanda, O.G., Juvencio, G.M., Nora, R.O., Cleotilde, J.R., Fortunata, S.T., & Oswaldo, R.M. (2013). Evaluating the degradation of the herbicides picloram and 2,4-D in a compartmentalized reactive barrier. Unpublished.

[CR59] Zhang, B.G. (2013). Culturable bacteria isolated from rhizosphere soil of Splendid achnatherum in the upper reach of Shule river. Unpublished.

[CR60] Zhao, L. (2014). The distribution of bacterial communities in Yanchang oil field in China site of Assam. Unpublished.

